# Influence of the image levels of distal femur on the measurement of tibial tubercle-trochlear groove distance—a comparative study

**DOI:** 10.1186/s13018-015-0323-4

**Published:** 2015-11-16

**Authors:** Li Yin, Cheng Chen, Xiaojun Duan, Bing Deng, Ran Xiong, Fuyou Wang, Liu Yang

**Affiliations:** Center for Joint Surgery, Southwest Hospital, The Third Military Medical University, 30 Gaotanyan St., Shapingba, Chongqing 400038 People’s Republic of China

**Keywords:** Knee, TT-TG distance, Image level, Femoral trochlea, CT, MRI

## Abstract

**Background:**

The purpose of the present study was to determine whether the image levels of the distal femur affected the measurement of the tibial tubercle-trochlear groove (TT-TG) distance.

**Methods:**

Thirty sets of computer tomography (CT) images and 30 sets of MR images of the knee were evaluated. The TT-TG distance was quantified at multiple image levels in 1.5-mm increments, covering the proximodistal range of the trochlear groove. The CT measurement was based on osseous landmarks; the magnetic resonance imaging (MRI) measurement was based on cartilaginous and osseous landmarks.

**Results:**

The average TT-TG distances measured with CT, with MRI based on cartilaginous landmarks, and with MRI based on osseous landmarks were 15.74 mm (SD 3.83 mm), 12.8 mm (SD 5.67 mm), and 12.36 mm (SD 5.58 mm), respectively. No significant difference was found across image levels in the CT measurement and the MRI measurement upon osseous landmarks (*P* = 0.64, *P* = 0.11); yet, the difference was significant in the MRI measurement upon cartilaginous landmarks (*P* < 0.01). Large deviation was found between levels in individual subjects in all the three sorts of measurement. The proximal levels were the most variable, while the mid levels were the least variable.

**Conclusions:**

Measurements of the TT-TG distance are not identical across the levels of the distal femur. Cautions should be taken when specific image slices were selected for evaluation.

## Introduction

The tibial tubercle-trochlear groove (TT-TG) distance is a widely used parameter to evaluate the lateral offset of the distal extensor mechanism. Increased TT-TG distance is usually deemed as a predisposing factor to patellar instability and anterior knee pain [1, 2]. For surgeons, the TT-TG distance of 20 mm or greater measured with computer tomography (CT) is considered pathological and is suggested as an indication for distal realignment procedures [2, 3].

The TT-TG distance is generally defined as the distance between the tip of the tibial tubercle and the bottom of the trochlear groove along the posterior tangent of the femoral condyles, measured on superimposed axial images of the knee [4]. However, the detailed measurement techniques are vaguely depicted in the literature and may be inconsistently executed across practices. Recent studies have revealed that many factors could be essentially influential to the TT-TG measurements, such as imaging modalities [5–7], knee flexion and rotation [8–12], and osseous or cartilaginous landmarks of choices [5, 13, 14]. Besides these, another important aspect remaining ambiguous in the literature is the proximodistal level of the image slice on which the anatomical landmarks of the distal femur (the deepest point of the trochlear groove and the posterior tangent of the femoral condyles) are identified for measurements. In previous studies, various image levels have been adopted without consensus, such as the most proximal slice on which a complete cartilaginous trochlea is seen [5, 15, 16], the most distal slice with full cartilage coverage of the trochlear groove [8], the slice with the maximal anteroposterior dimension of the femoral condyles [1, 17, 18], the slice on which the femur presents as the best Roman arch [11, 13, 14], and the slice with the deepest yet still fully recognizable trochlear groove [19]. Given that the shape of the distal femur varies across axial levels, the different selection of image slices may lead to deviation in the measured TT-TG distance. However, this influence has not yet been investigated and thus is still not well understood.

The objective of the current study was to quantify the TT-TG distances with respect to different levels of the distal femur with both CT and magnetic resonance imaging (MRI) measurements. We hypothesized that the TT-TG distance might not be identically measured throughout the proximodistal range of the femoral trochlea.

## Materials and method

### Subjects

From a knee imaging repository in our laboratory, we retrospectively selected 30 subjects (30 knees) with CT scans and another 30 subjects (30 knees) with axial MRI scans in the knee. These subjects were recruited for image collection between 2009 and 2012 for research purpose. All subjects were healthy and had been excluded for deformity, injury, or surgery history in the lower limbs. The CT group consisted of 18 males and 12 females, 15 left knees and 15 right knees, with an average age of 26.4 years (SD 4.8 years); the MRI group consisted of 11 males and 19 females, 7 left knees and 23 right knees, with an average age of 27.3 years (SD 5.3 years). The sample size was estimated by a priori power analysis using G-Power (version 3.1.9.2, http://www.gpower.hhu.de), which suggested a sample size of 20 (*α* = 0.05, effect size = 0.25) to provide a power of 95 %. The current study has been approved by the ethic committee of the authors’ institution (Southwest Hospital). All subjects in the imaging repository had been informed that their image data would be utilized for scientific research as well as publication and had provided informed consent prior to the image collection.

### Imaging

The CT scans were performed using a 16-slice spiral scanner (SOMATOM, Siemens, Munich, Germany) with the knee in extension. The range of the distal femur through the proximal tibia was covered. Images were obtained with use of 0.625 mm of slice increment, 110 kV 230 mAs, and in-plane resolution of 512 × 512. The MRI scans were performed on a 3.0T scanner (GE Healthcare, Milwaukee, USA) with the knee in extension, using an eight-element knee coil and a 3D fat-suppressed fast spoiled gradient-recalled echo sequence (TR: 14.5 ms; TE: 2.8 ms; slice increment: 1 mm; matrix: 320 × 320).

The original CT and MRI data were imported into Mimics (version 14.1, Materialise, Leuven, Belgium). The imported images were first reconstructed into volume and then resliced to create a new axial image set with the slice increment of 1.5 mm.

### Measurements of the TT-TG distance

**Fig. 1:**
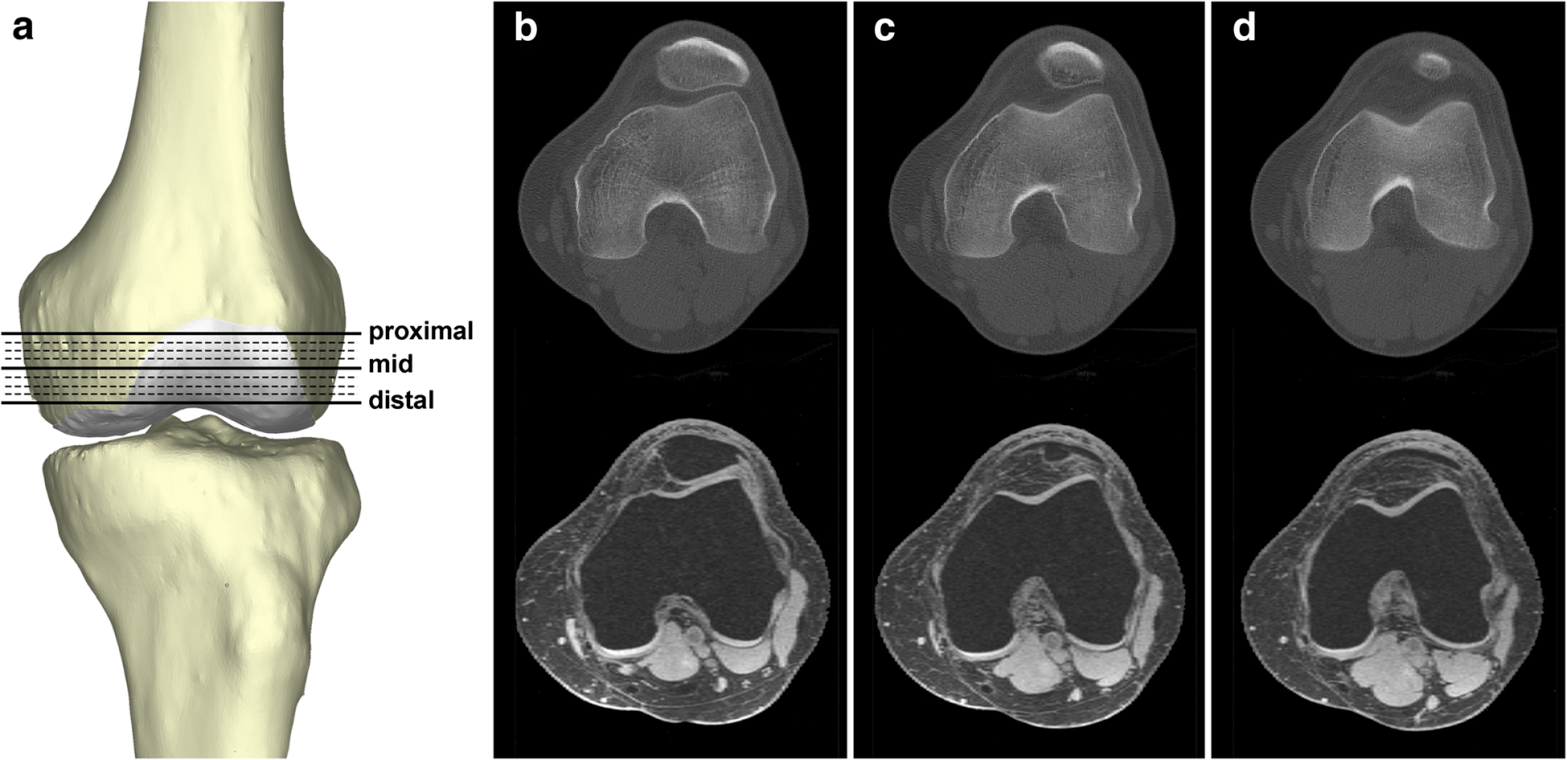
The definition of the trochlear range. **a** Resliced axial images from the proximal entrance to the distal end of the femoral trochlea in 1.5-mm increments were selected for the evaluation of the tibial tubercle-trochlear groove distance. The trochlear groove and posterior condyles exhibit distinct shapes at different levels of the distal femur. **b** Proximal level. **c** Mid level. **d** Distal level

For each resliced CT or MRI image set, the consecutive image sequence from the level showing the most proximal groove point of the trochlea (referred to as the trochlear entrance hereafter) to the most distal level on which a complete trochlea remained identifiable was picked and exported, named the trochlear range. The numbers of the image slices within the trochlear range varied from 10 to 14, depending on the individual knee size (Fig. 1). One single image on which the tibial tubercle presented the most prominent shape was also chosen and exported. All selected images were saved in BMP format for further evaluation. The TT-TG distance would be quantified on each image slice in the trochlear range, representing the measurements with respect to different levels of the distal femur in a 1.5-mm increment.

**Fig. 2:**
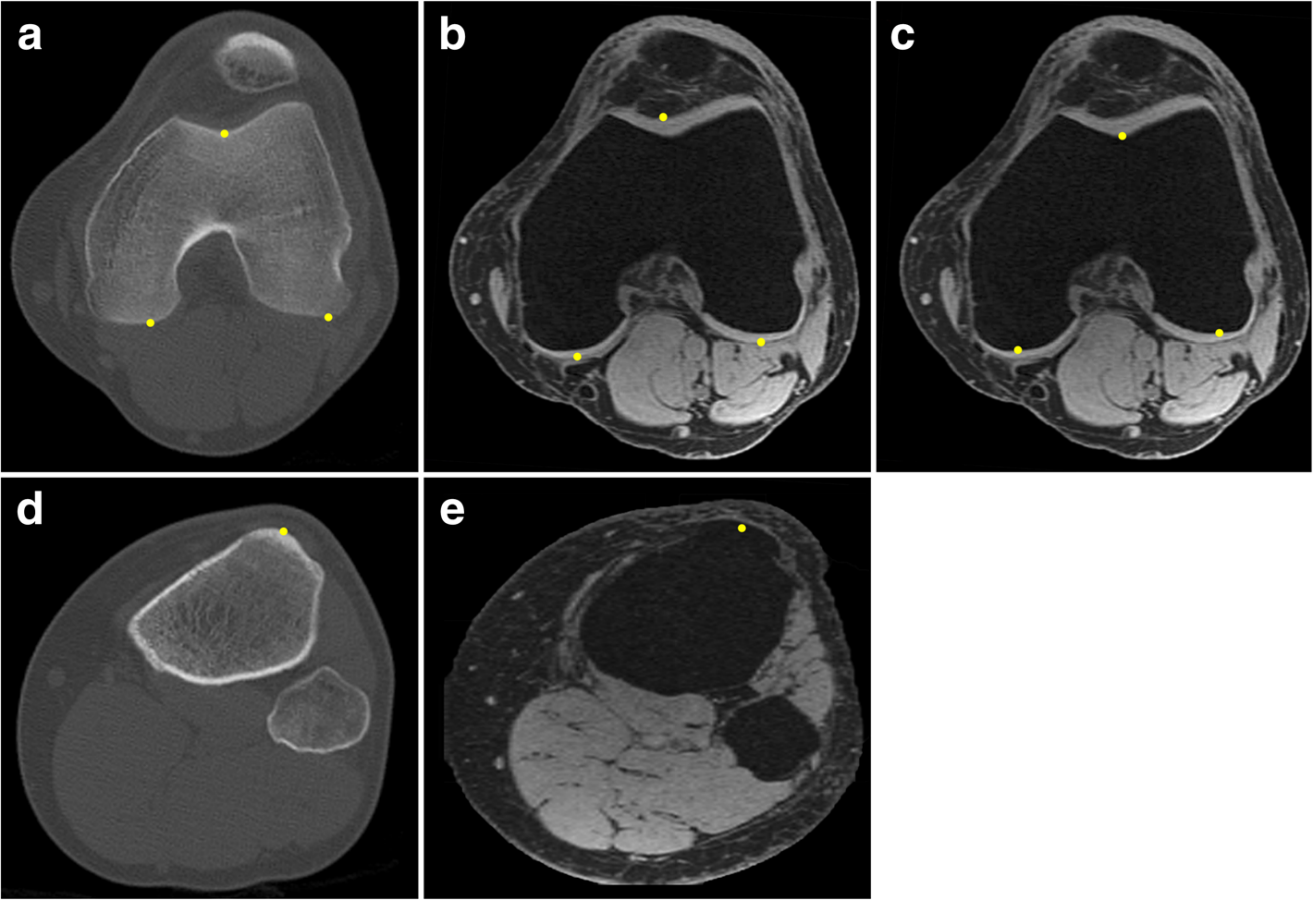
Markings of the anatomical landmarks for the measurement of the tibial tubercle-trochlear groove distance. The deepest point of the trochlear groove, the points of tangency of the posterior femoral condyles, and the most prominent point of the tibial tubercle are identified and marked. **a** CT of the distal femur. **b** MRI of the distal femur with cartilaginous landmarks. **c** MRI of the distal femur with osseous landmarks. **d** CT of the proximal tibia. **e** MRI of the proximal tibia

**Fig. 3:**
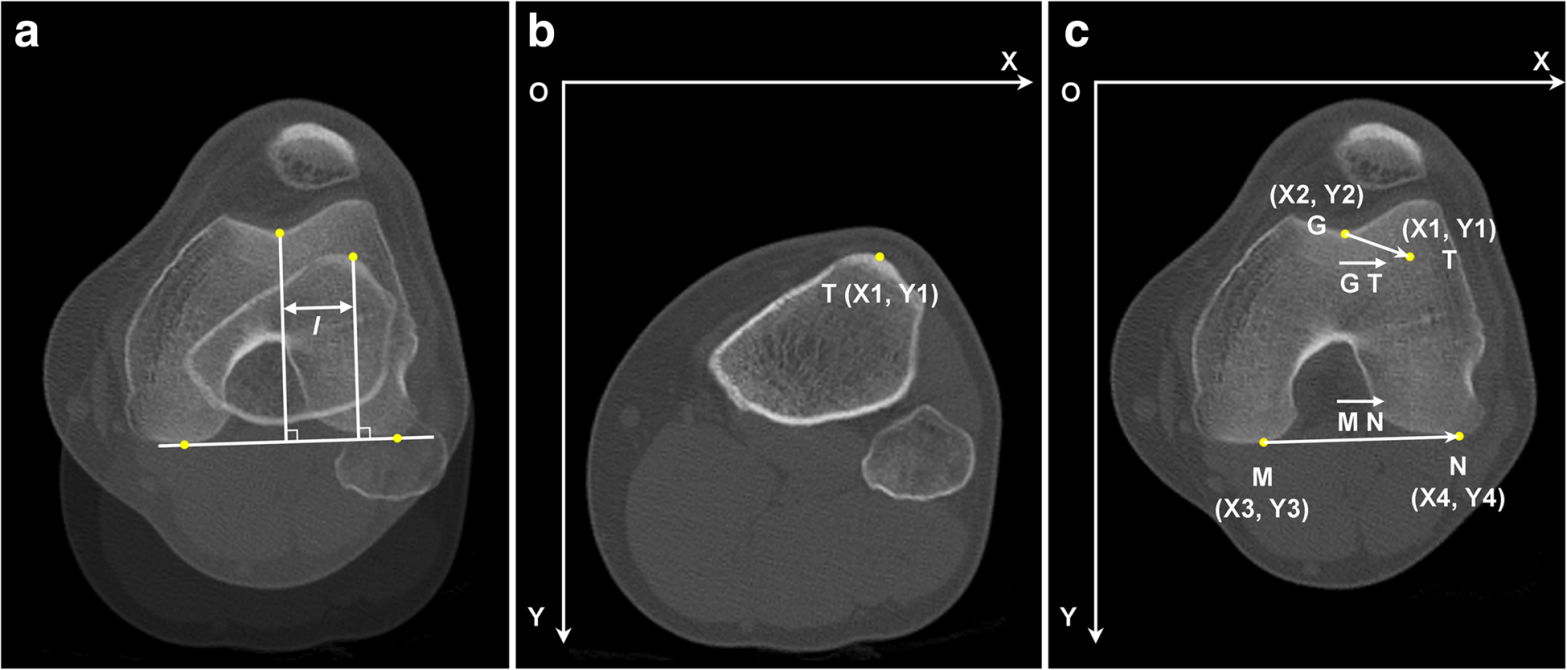
The measurements of the tibial tubercle-trochlear groove (TT-TG) distance. **a** The prevailing “two-slice measurement” method. The levels of the tibial tubercle and the trochlear groove are superimposed onto one image; the TT-TG distance (*l*) is measured as the distance between the two perpendiculars to the tangent of the posterior femoral condyles which also pass through the tip of the tibial tubercle and the deepest point of the trochlear groove, respectively. **b**, **c** The vector calculation used in the current study. The tip of the tibial tubercle was termed *T*, the bottom of the trochlear groove was termed *G*, and the points of tangency of the posterior femoral condyles were termed *M* and *N*. The TT-TG distance can be calculated upon the vectors of $\overrightarrow{GT}$ and $\overrightarrow{MN}$, by $\left|\frac{\overrightarrow{GT}\cdot \overrightarrow{MN}}{\left|\overrightarrow{MN}\right|}\right|$

Three categories of TT-TG distance were independently quantified in the current study: measurements with CT, measurements with MRI based on cartilaginous landmarks, and measurements with MRI based on osseous landmarks, named the CT group, cartilaginous-MRI group, and osseous-MRI group, respectively. Using Image J (Version 1.47, Http://imagej.nih.gov/ij), the deepest point in the trochlear groove as well as the two points of tangency on the posterior femoral condyles were identified and marked on each image within the trochlear range. Similarly, the most prominent point of the tibial tubercle was also marked (Fig. 2). The TT-TG distance was computed using a custom-developed script based on vector calculation. The marked points were first recognized, and their coordinates were recorded; the TT-TG distance was computed as the distance between the marked points of the trochlear groove and the tibial tubercle projected onto the line connecting the two points of tangency of the posterior femoral condyles (Fig. 3). The measurements were carried out by an orthopedic surgeon with 7 years of experience (LY).

### Reliability

Intra- and inter-rater reliability of the measurements was assessed by repeatedly measuring a selection of images. For each subject, the 2nd, the 6th, and the 10th slices of the trochlear range counted in the proximal-to-distal direction (the slices 1.5, 7.5, and 13.5 mm distal to the trochlear entrance, respectively) were picked for repeated measurements. These three slices, respectively, represented the proximal, mid, and distal levels of the trochlear range. For each subject, two orthopedic surgeons (LY and RX) with 7 and 8 years of experience, respectively, independently marked the three selected images with a 2-week interval. As the reliability tests primarily focused on the systematic error induced by the image levels on the femoral side, all measurements of one subject used the same image with marked tibial tubercle to eliminate noises from the tibial side. The images underwent the same processes mentioned above to calculate the TT-TG distances. Intra- and inter-rater reliability was evaluated using the intra-class correlation coefficient (ICC), with the absolute agreement checked by a two-way random model.

### Data analysis

For each subject, the TT-TG distance measured at every level of the distal femur was quantified; the mean value across all levels was then calculated. Based on these data, two sorts of deviations were subsequently calculated in each subject: the deviation between the maximal and the minimal TT-TG distances across levels, which indicated the range of the variance derived from levels, and the deviation of the TT-TG distance at each level to the mean value across levels, which indicated the level-specific variance. After data normality was checked using the Shapiro-Wilk method, the influence of the image levels on the TT-TG distance was characterized with one-way repeated measures ANOVA. Furthermore, the TT-TG distances of the cartilaginous-MRI group and osseous-MRI group at the same level were compared utilizing paired-sample *t* tests. All statistical calculations in the current study were performed in SPSS 18.0 (IBM, Armonk, USA). The significance level was set as *P* < 0.05.

## Results

The TT-TG distances at all levels throughout the trochlear range were successfully quantified for all subjects in the CT, cartilaginous-MRI, and osseous-MRI groups (Table 1). Repeated measurements revealed high intra- and inter-rater ICCs for the three groups at all the three selected levels, indicating that the measurements were reliable (Tables 2 and 3).

**Table 1:** Summary of the TT-TG distances in the CT, cartilaginous-MRI, and osseous-MRI groups

	TT-TG distance (mm)		Maximal deviation across levels (mm)	
	Mean (SD)	Range	Mean (SD)	Range
CT	15.75 (3.84)	8.8–23.76	2.6 (1.14)	1.01–5.26
Cartilaginous-MRI	12.8 (5.67)	0.75–22.08	2.73 (1.25)	0.54–5.74
Osseous-MRI	12.36 (5.58)	0.85–22.04	2.11 (0.95)	0.77–4.83

**Table 2:** Intra-class correlation coefficient and 95 % confidence interval for the intra-rater reliability

	Proximal level	Mid level	Distal level
CT	0.987 (0.972–0.994)	0.988 (0.972–0.995)	0.990 (0.975–0.996)
Cartilaginous-MRI	0.997 (0.993–0.998)	0.998 (0.995–0.999)	0.997 (0.995–0.999)
Osseous-MRI	0.996 (0.993–0.998)	0.997 (0.994–0.999)	0.998 (0.996–0.999)

The table shows the lower values of the two raters

**Table 3:** Intra-class correlation coefficient and 95 % confidence interval for the inter-rater reliability

	Proximal level	Mid level	Distal level
CT	0.978 (0.953–0.989)	0.987 (0.973–0.994)	0.984 (0.966–0.992)
Cartilaginous-MRI	0.986 (0.940–0.995)	0.991 (0.905–0.998)	0.993 (0.968–0.997)
Osseous-MRI	0.995 (0.990–0.998)	0.997 (0.993–0.998)	0.997 (0.991–0.999)

For each rater, the mean of the two measurements was used for calculation

**Fig. 4:**
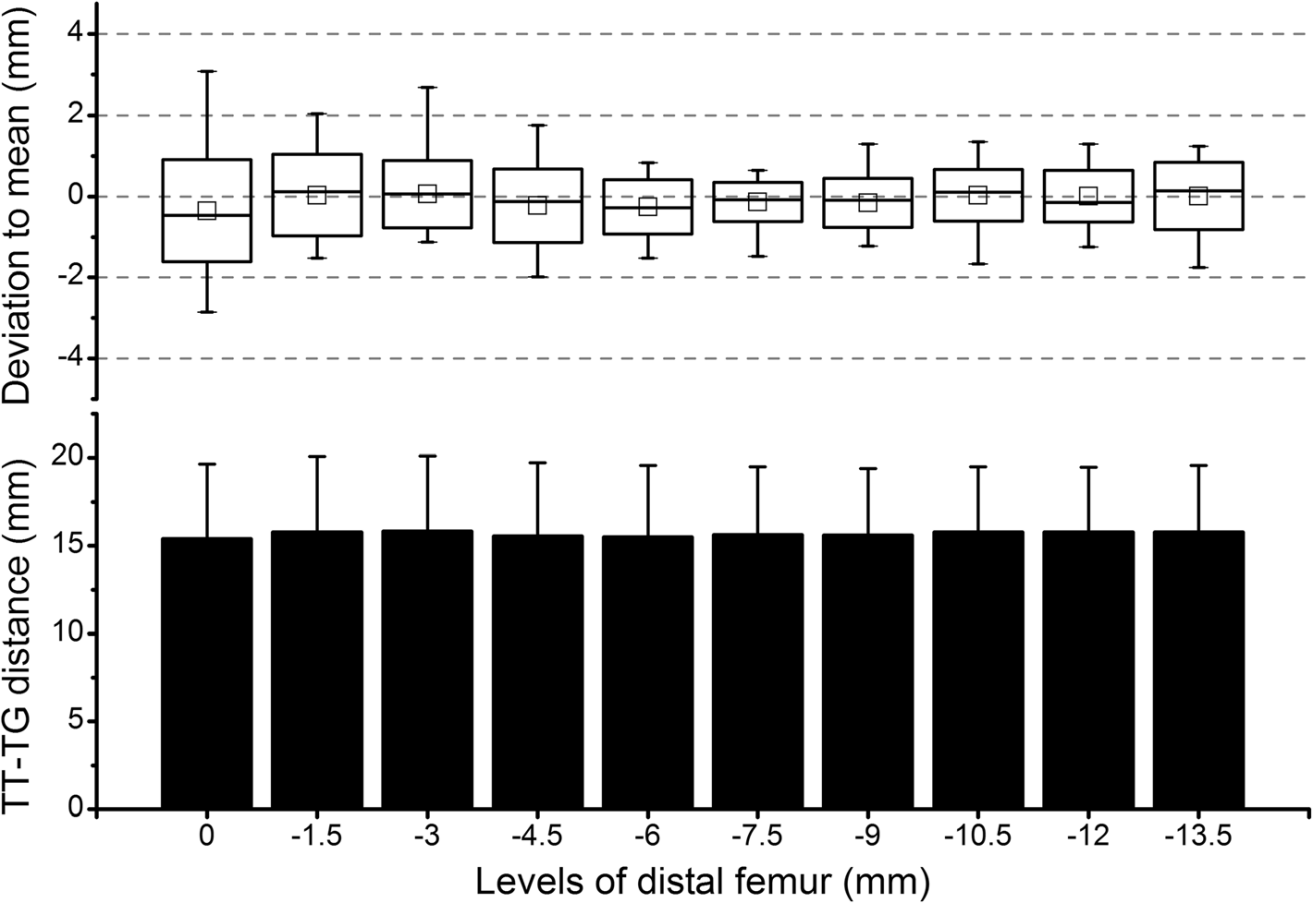
Results of the CT group. *Column plot* shows the tibial tubercle-trochlear groove (TT-TG) distance of all 30 subjects at various levels of the distal femur. On the *X*-axis, the level of the trochlear entrance was defined as 0; the distal to the trochlear entrance was defined as negative. *Box plot* shows the deviation of the TT-TG distance at each level in relation to the average value across levels. The *square in the box* indicates the mean, the *line* indicates the median, the *upper and lower ends of the box* indicate one standard deviation, and the *whiskers* indicate the range of all values

Because the subjects have different numbers of levels in the trochlear range (10 to 14, average 12.1) due to individual knee size, only the first 10 levels counted in the proximal-to-distal direction which were common across subjects were selected for the repeated measures ANOVA. In the CT group, the levels of the distal femur did not have a significant effect on the TT-TG distance (*P* = 0.37) (Fig. 4). The deviation to the mean value was close to 0 mm throughout the levels; however, a relatively large range was observed. The values were the most variable within the interval of 0 through 4.5 mm below the trochlear entrance and the least variable within the interval of 6 through 9 mm below the trochlear entrance (Fig. 4).

**Fig. 5:**
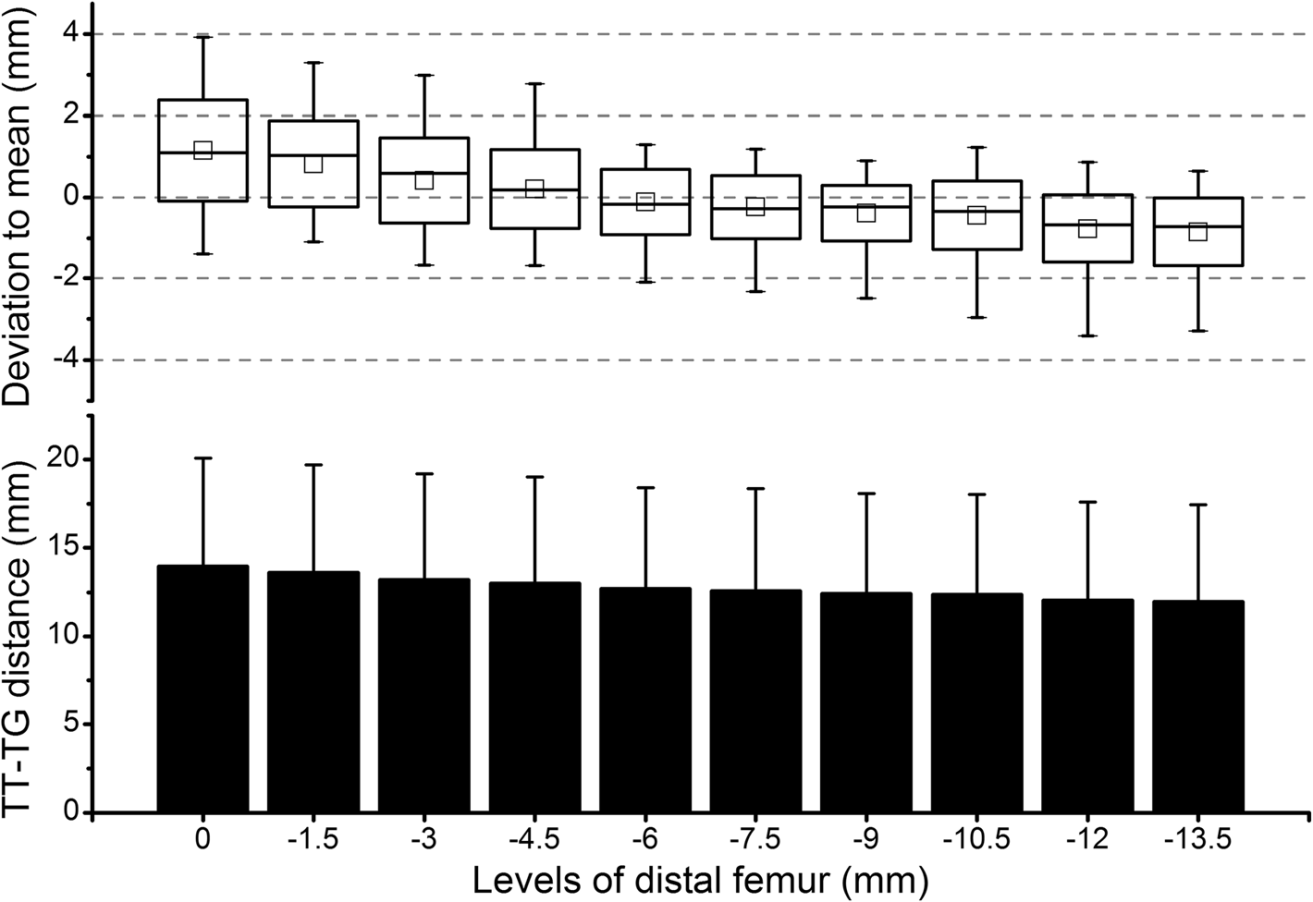
Results of the cartilaginous-MRI group. *Column plot* shows the tibial tubercle-trochlear groove (TT-TG) distance of all 30 subjects at various levels of the distal femur. On the *X*-axis, the level of the trochlear entrance was defined as 0; the distal to the trochlear entrance was defined as negative. *Box plot* shows the deviation of the TT-TG distance at each level in relation to the average value across levels. The *square in the box* indicates the mean, the *line* indicates the median, the *upper and lower ends of the box* indicate one standard deviation, and the *whiskers* indicate the range of all values

In the cartilaginous-MRI group, the levels of the distal femur had a significant effect on the TT-TG distance (*P* < 0.01). The value tended to decline with the distalization of levels (Fig. 5). Correspondingly, the deviation to the mean value also decreased from positive at proximal levels to negative at distal levels. Similar to the CT group, a large range of the deviation was observed. The deviation was most variable within the interval of 0 through 4.5 mm below the trochlear entrance and least variable within the interval of 6 through 9 mm below the trochlear entrance (Fig. 5).

**Fig. 6:**
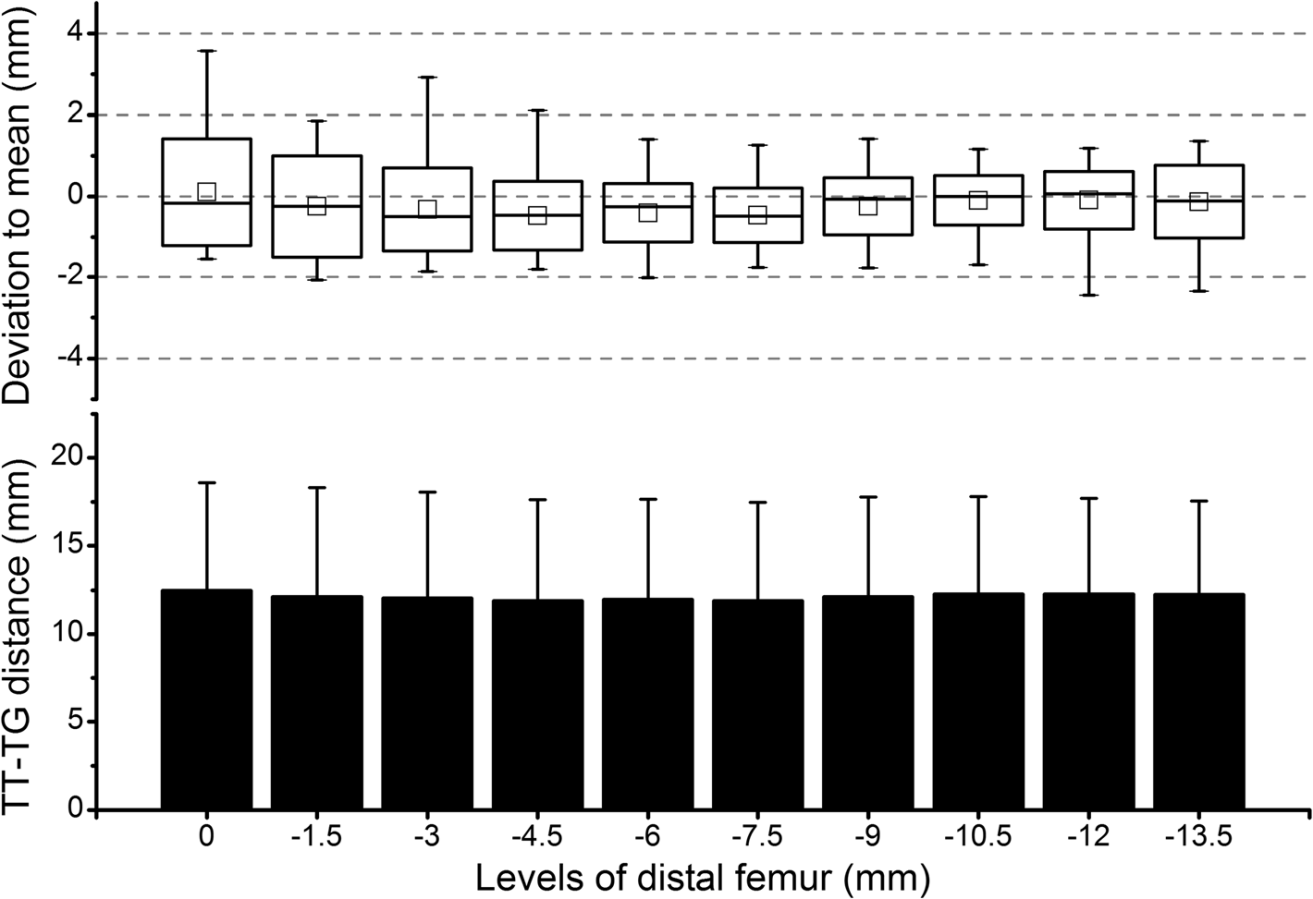
Results of the osseous-MRI group. *Column plot* shows the tibial tubercle-trochlear groove (TT-TG) distance of all 30 subjects at various levels of the distal femur. On the *X*-axis, the level of the trochlear entrance was defined as 0; the distal to the trochlear entrance was defined as negative. *Box plot* shows the deviation of the TT-TG distance at each level in relation to the average value across levels. The *square in the box* indicates the mean, the *line* indicates the median, the *upper and lower ends of the box* indicate one standard deviation, and the *whiskers* indicate the range of all values

In the osseous-MRI group, the levels of the distal femur did not have a significant effect on the TT-TG distance (*P* = 0.11) (Fig. 6). Similar to the CT group, the deviation to the mean value was close to 0 mm throughout levels, with a relatively large variation within the interval of 0 through 4.5 mm below the trochlear entrance and a small variation within the interval of 6 through 10.5 mm below the trochlear entrance (Fig. 6).

**Fig. 7:**
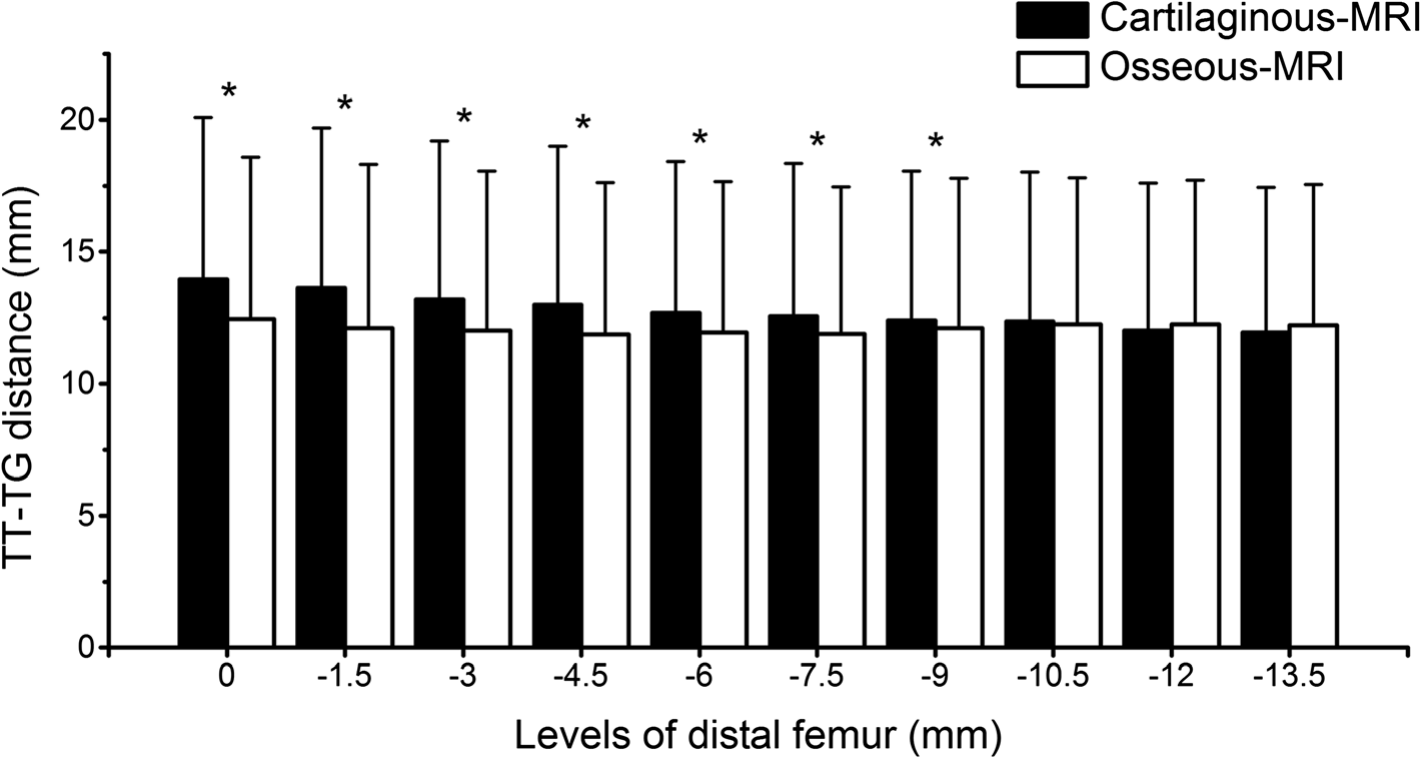
Comparison of the TT-TG distances between the cartilaginous-MRI group and the osseous-MRI group. *Asterisk* indicates significant difference between groups

For the same reason mentioned above, only the first 10 levels in the trochlear range were picked for the paired-sample *t* tests between the osseous-MRI group and cartilaginous-MRI group. Except for the 8th, the 9th, and the 10th levels (10.5, 12, and 13.5 mm below the trochlear entrance, respectively), significant differences were observed between groups (*P* < 0.05) (Fig. 7).

## Discussion

Based on reliable measurements of the TT-TG distance across multiple image levels of the distal femur, we found that the levels had a significant influence on the results when cartilaginous landmarks were used. The influence was not significant when the values were based on osseous landmarks, with both MRI and CT measurements. However, large deviations of the TT-TG distance between levels could still be observed in some individual subjects. These findings supported our hypothesis that the image levels of the distal femur might affect the results of the TT-TG measurements.

The examination as well as the measurement techniques of the TT-TG distance has been evolving over the past decades. The TT-TG distance was initially described by Goutallier et al., who suggested the use of an axial radiograph taken at 30° of knee flexion with neutral axial rotation for the measurement [4]. With the development of CT, which was able to provide high-quality axial images, CT measurements prevailed in the following years. As suggested by Dejour et al., the CT scans were conducted with the knee in extension instead of 30° of flexion [2]. Early CT measurements were performed upon the so-called maximum intensity projection, which was reformatted from the original cross-sectional images in the way that all anatomical structures were superimposed [2, 7]. As this method had been criticized for sometimes difficultly detectable anatomical structures and low intra- and inter-rater reliability [20], new measurement approaches had been proposed. These new methods were commonly featured with the “two-slice measurement,” in which the bottom of the trochlear groove and the posterior tangent of the femoral condyles were identified based on one image slice selected from the range of the distal femur; the tip of the tibial tubercle was similarly identified on another image slice from the proximal tibia; measurements were conducted based on the two separate images rather than the all-in-one superimposed geometries [21]. The use of MRI has been prevailing in TT-TG measurement in recent years, owing to its additional benefits such as low radiation and superior soft tissue visualization. The two-slice measurement was still maintained as the major technique in the MRI measurements, as shown in previous studies [5, 14, 16, 19, 22]. Nevertheless, the disadvantage of the two-slice measurement has been noted by previous author. Lustig et al. inferred that the axial views of the knee might be a contributor to the measurement bias observed in their study, since choosing two identical axial views of exactly the same level at two separate measurements was difficult to achieve. This issue not only existed in the processes of measurement but also existed in the CT or MRI scanning when the axial images were generated [23].

As currently documented in the literature, various criterions have been held for the selection of image levels. These differing criterions may induce errors to the measurements, leading to less comparable results between studies. For example, according to our data, the most proximal and the most distal slices showing a complete cartilaginous trochlea had an average vertical distance of 16 mm; the average TT-TG distances measured based on cartilaginous landmarks at these two levels were 13.95 and 11.95 mm, respectively, which were significantly different (*P* < 0.001). Although no significant difference in the measurements across levels was found when osseous landmarks were used, notable deviations between values as high as 5 mm could still be observed in some individual subjects. Therefore, a standardized and consistent criterion for the selection of image levels would be beneficial for the TT-TG measurements. According to our results, the interval of 0 through 4.5 mm below the trochlear entrance (approximating the proximal levels) had the greatest deviation to the mean TT-TG distance; in contrast, the interval of 6 through 9 mm below the trochlear entrance (approximating the mid levels) was the least variable. From this point of view, the latter may be a favorable range for the TT-TG measurements; yet, cautions should be taken when the former is employed.

We also noted an essential mismatch in the TT-TG distances of the cartilaginous-MRI group and the osseous-MRI group. From the proximal end to the distal end, the osseous landmarks lead to values closely around the average, yet the cartilaginous landmarks lead to a stable decline in the values. The cartilaginous-MRI group came with significantly higher values than the osseous-MRI group at most levels. The inconsistency of the cartilaginous and osseous measurements has also been reported by previous authors [5, 13, 14]. These findings may be explained by the mismatch in the shapes of the cartilaginous and osseous trochlea. As revealed in previous studies, the thickness of the cartilage was unevenly distributed in the trochlear groove, which resulted in an offset of the deepest points of the cartilaginous and the osseous trochlea, further leading to deviations in the dependent TT-TG distances [24, 25].

We acknowledge the limitations in the current study. The CT and MRI groups involved different subjects; with the same subjects involved, the results could have been more confident to show the potential difference between imaging modalities. Besides, the subjects involved were all healthy, with normal patellofemoral development. However, the TT-TG measurements were frequently conducted in patients with dysplastic trochlea. One can expect that the results observed in our study might be different when it comes to dysplastic trochlea. Moreover, the subjects were all drawn from Asian population; future investigations based on Caucasian population are still in favor to provide more complete data.

## Conclusion

The image levels of the distal femur have significant influence on the TT-TG distance measured based on cartilaginous landmarks, but not on those based on osseous landmarks; however, notable deviation between levels exists in some individual subjects for all sorts of measurements.

### Clinical relevance

Cautions should be taken by clinicians when image slices are selected for the measurement of the TT-TG distance. The interval of 6 through 9 mm below the trochlear entrance (approximating the mid levels) of the femoral trochlea may be a favorable range.

## Abbreviations

CT: computer tomography
ICC: intra-class correlation coefficient
MRI: magnetic resonance imaging
TT-TG: tibial tubercle-trochlear groove
